# The impact of heart failure and bleeding risk on clinical outcomes in patients after percutaneous coronary intervention

**DOI:** 10.1186/s12872-025-05429-6

**Published:** 2025-12-19

**Authors:** Yoshiyuki Yazaki, Yoshihisa Nakagawa, Ken Kozuma, Raisuke Iijima, Anna Tsutsui, Yoshitaka Murakami, Masayuki Fukuzawa, Satoru Abe, Go Kato, Hidehiko Hara, Masato Nakamura

**Affiliations:** 1 Division of Cardiovascular Medicine, Toho University Ohashi Medical Centre, 2-22-36, Ohashi, Meguro-ku, Tokyo, 153-8515 Japan; 2 Department of Cardiology, Misato Central General Hospital, Saitama, Japan; 3 Department of Cardiovascular Medicine, Shiga University of Medical Science, Otsu, Japan; 4 Division of Cardiology, Department of Internal Medicine, Teikyo University, Tokyo, Japan; 5 Department of Medical Statistics, School of Medicine, Toho University, Tokyo, Japan; 6 Primary Medical Science Department, Daiichi Sankyo Co., Ltd, Tokyo, Japan; 7 Division of Minimally Invasive Treatment in Cardiovascular Medicine, Toho University Ohashi Medical Centre, Tokyo, Japan

**Keywords:** Heart failure, Percutaneous coronary intervention, Major bleeding, Major adverse cardiac and cerebrovascular events, High bleeding risk

## Abstract

**Background:**

In recent years, revascularization for ischaemic heart failure (HF) has received increasing attention, and percutaneous coronary intervention (PCI) has been positioned as a high-risk procedure. However, the risk of major bleeding and major adverse cardiac and cerebrovascular events (MACCE) after PCI for patients with ischaemic HF has not been adequately evaluated.

**Methods:**

HF patients were selected from the PENDULUM (Platelet rEactivity in patieNts with DrUg eLUting stent and balancing risk of bleeding and ischaemic event) registry to compare the efficacy and safety of PCI in HF and non-HF patients. In addition, the impact of high bleeding risk (HBR) defined as BARC 3 and 5 was evaluated in these patients.

**Results:**

A total of 6266 patients were included; 16% (*n* = 1006) had a history of HF at enrolment. Patients with HF showed a higher MACCE rate than those without HF (18.4% versus 7.7%; adjusted hazard ratio [HR] = 1.59, 95%CI, 1.30–1.93; *p* < 0.001) and a significant difference in major bleeding (9.3% versus 3.3%; adjusted HR = 1.74, 95%CI, 1.30–2.33; *p* < 0.001). HBR was significantly more associated with the MACCE and major bleeding rates than non-HBR, regardless of HF status (adjusted HR = 2.93, 95% CI: 2.33–3.69, *p* < 0.001, p for interaction = 0.913, adjusted HR = 2.67, 95% CI: 1.89–3.76, *p* < 0.001, p for interaction = 0.465, respectively).

**Conclusions:**

After contemporary PCI, HF and HBR were independently associated with a higher risk of MACCE and bleeding. HF patients after PCI require more careful management to improve prognosis, particularly with HBR factors.

**Graphical Abstract:**

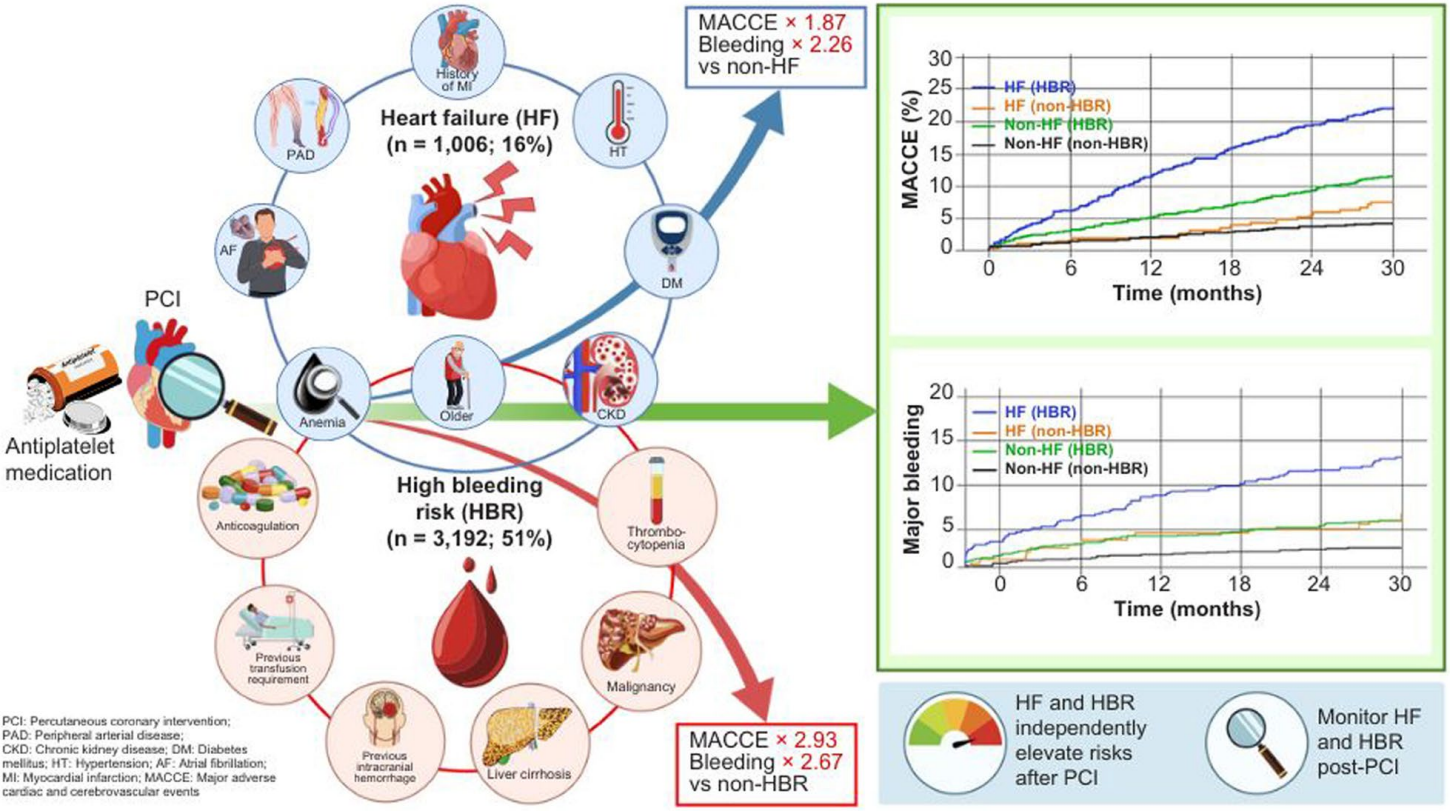

**Supplementary Information:**

The online version contains supplementary material available at 10.1186/s12872-025-05429-6.

## Introduction

In recent years, cardiovascular events after percutaneous coronary intervention (PCI) have been decreasing as a result of technological advances and the introduction of optimal medical treatment [1, 2]. However, with regard to medical management after PCI, a causal relationship between bleeding complications and subsequent increased cardiovascular mortality has been suggested, and strategies to prevent bleeding events are attracting attention [3, 4]. Therefore, the Academic Research Consortium – high bleeding risk (ARC-HBR) has strongly advocated for general bleeding risk assessment after PCI [5, 6]. Indeed, the PENDULUM (Platelet rEactivity in patieNts with DrUg eLUting stent and balancing risk of bleeding and ischaemic event) registry, a prospective, multicentre study of Japanese patients who underwent PCI, demonstrated that HBR patients who met the ARC-HBR criteria developed significantly more major bleeding [7, 8]. In addition, a post hoc analysis of this registry study reported that HBR patients had a 2.8-fold higher rate of thrombotic/ischaemic events than non-HBR patients [9]. These findings suggest that the management of HBR patients is complex, balancing bleeding and ischaemic events.

Coronary artery disease is one of the most common comorbidities of heart failure (HF), and ischaemic heart failure is associated with a high incidence of cardiovascular events and a poor prognosis [10, 11]. Though the role of revascularization for ischaemic heart failure remains debatable, the use of PCI in HF patients with reduced ejection fraction has increased markedly, and such patients are widely recognized as a high-risk group for cardiovascular events [12, 13]. In addition, although not included as a risk factor in ARC-HBR, HF patients may have a higher bleeding risk due to their many comorbidities and medications [5, 6]. Thus, post-PCI management of patients with HF has similar considerations to those of patients with HBR and should be considered thoughtfully. However, whether the presence of HBR has any prognostic impact on bleeding events or cardiovascular events in the high-risk group after PCI in patients with HF is unclear. Therefore, the purpose of this study was to evaluate the impact of HBR on cardiovascular and bleeding events in HF patients after PCI as a post hoc analysis of the PENDULUM registry [7, 8]. In addition, the presence of HF was also evaluated for its effect on the relationship between HBR and the incidence of cardiovascular and bleeding events.

## Methods

### Study population and design

Full inclusion and exclusion criteria for the PENDULUM registry have been reported previously [7]. In brief, patients aged 20 years and older at the time of informed consent, indicated for PCI with a second-generation drug-eluting stent, and administered antiplatelet drugs were enrolled. HF was defined as a history of HF at enrolment, and the diagnosis was made by the attending physician based on congestive symptoms and laboratory findings. Baseline left ventricular ejection fraction was recorded when available, but it was not an inclusion requirement for the PENDULUM registry. For the post hoc criterion analysis, enrolled patients were previously stratified into HBR and non-HBR groups according to ARC-HBR criteria [8]. In this study, platelet reactivity was assessed using P2Y_12_ reaction unit (PRU) values between 12 and 48 h after PCI, and patients were divided into two groups, the high platelet reactivity (HPR) and the non-HPR groups, with 208 as the cut-off value [14]. 

### Clinical outcomes

The primary endpoints were major adverse cardiac and cerebrovascular events (MACCE; the standard clinical definition comprising all-cause death, non-fatal myocardial infarction (MI), non-fatal stroke, and stent thrombosis) and major bleeding, as defined by BARC (Bleeding Academic Research Consortium) type 3 or 5 bleeding after the index PCI [15]. The secondary endpoints were the individual components of MACCE.

### Statistical analysis

Baseline characteristics are described by frequencies and percentages for categorical variables and mean and standard deviation (SD) values for continuous variables. Fisher’s exact test was used for the comparison of categorical variables, and Student’s *t*-test was used to compare continuous variables. Cumulative incidence rates among comparison groups were calculated, and Kaplan-Meier curves and Cox proportional hazards models were used for the group comparison.

Two types of multivariate Cox proportional hazards models were used to estimate hazard ratios (HRs) with 95% confidence intervals (CIs). In the first Cox model, factors associated with MACCE and major bleeding events in patients with or without HF were examined. To adjust for confounders, the following clinically relevant factors were selected as in previous study [3, 7] : sex, age, body weight, diabetes mellitus, ACS (acute coronary syndrome)/non-ACS, presence of peripheral arterial disease (PAD), chronic kidney disease, anaemia, and history of stroke or cerebral haemorrhage for MACCE; and sex, body weight, ACS/non-ACS, presence of PAD, chronic kidney disease, anaemia, malignant tumour, and anticoagulation at discharge for major bleeding events. In the second Cox model, the relationship between HF/HBR and outcomes was assessed by HRs with 95% CIs based on multivariate Cox proportional hazards models. In the model, factors associated with MACCE and major bleeding events in patients with or without HF and HBR, and the interaction of HF and HBR, were examined. Because HBR was included in this model, variables used for HBR evaluation were not included. Thus, the following clinically relevant factors were selected to adjust for confounding: sex, diabetes mellitus, ACS/non-ACS, and presence of PAD for MACCE; and sex, ACS/non-ACS, and presence of PAD for major bleeding events.

All statistical analyses were performed using SAS, version 9.4 (SAS Institute, Cary, NC, USA). All tests were two-sided, and *P* = 0.05 was considered significant.

## Results

### Patient characteristics

A total of 6266 patients were included in this post hoc study, of whom 1006 had a history of HF (755 in the HBR group and 251 in the non-HBR group). Demographically, patients with HF were significantly more likely to be older and have diabetes mellitus, hypertension, a history of myocardial infarction, PAD, and atrial fibrillation (Table 1). Concerning laboratory values, patients with HF were significantly more likely to have lower haemoglobin, estimated glomerular filtration rates, and left ventricular ejection fraction than those without HF (Table 1).

**Table 1:** Baseline characteristics of patients with and without heart failure

	Total (*n* = 6,266)	HF (*n* = 1,006)	Non-HF (*n* = 5,260)	*P* value
Age (y), mean (SD)	70.0 (10.7)	72.8 (11.1)	69.5 (10.6)	< 0.001
≥75 y	2,325 (37.1)	497 (49.4)	1,828 (34.8)	< 0.001
Body weight (kg), mean (SD)	63.9 (12.6)	61.4 (13.2)	64.4 (12.4)	< 0.001
≤50 kg	794 (12.7)	189 (18.8)	605 (11.5)	< 0.001
Body mass index (kg/m2), mean (SD)	24.21 (3.62)	23.72 (3.96)	24.31 (3.54)	< 0.001
Hypertension	5,188 (82.8)	895 (89.0)	4,293 (81.6)	< 0.001
Hyperlipidaemia	4,926 (78.6)	762 (75.7)	4,164 (79.2)	0.017
Diabetes mellitus	2,771 (44.2)	567 (56.4)	2,204 (41.9)	< 0.001
Current cigarette smoking	1,327 (21.2)	181 (18.0)	1,146 (21.8)	0.007
Peripheral arterial disease	438 (7.0)	106 (10.5)	332 (6.3)	< 0.001
Atrial fibrillation	539 (8.6)	192 (19.1)	347 (6.6)	< 0.001
Malignancy	389 (6.2)	78 (7.8)	311 (5.9)	0.032
Previous MI	1,575 (25.1)	394 (39.2)	1,181 (22.5)	< 0.001
Previous PCI	2,566 (41.0)	470 (46.7)	2,096 (39.8)	< 0.001
Previous CABG	265 (4.2)	85 (8.4)	180 (3.4)	< 0.001
History of ischaemic stroke	657 (10.5)	153 (15.2)	504 (9.6)	< 0.001
History of cerebral haemorrhage	124 (2.0)	25 (2.5)	99 (1.9)	0.216
History of renal insufficiency	1,114 (17.8)	346 (34.4)	768 (14.6)	< 0.001
Clinical presentation				
ACS	2,015 (32.2)	261 (25.9)	1,754 (33.3)	< 0.001
Unstable angina	790 (12.6)	83 (8.3)	707 (13.4)	0.003
Non-STEMI	323 (5.2)	59 (5.9)	264 (5.0)	-
STEMI	908 (14.5)	119 (11.8)	789 (15.0)	-
Hb (g/dL), Mean (SD)	13.30 (2.04)	12.43 (2.47)	13.46 (1.90)	< 0.001
Hb: <11 g/dL (both)	727 (11.6)	250 (24.9)	477 (9.1)	< 0.001
eGFR (mL/min/1.73 m^2^), Mean (SD)	61.25 (27.61)	50.36 (33.37)	63.34 (25.84)	< 0.001
eGFR: <60 mL/min/1.73 m^2^	2,690 (42.9)	631 (62.7)	2,059 (39.1)	< 0.001
Angiographic features				-
No. of diseased vessels				-
1	3,165 (50.5)	419 (41.7)	2,746 (52.2)	< 0.001
2	1,864 (29.7)	316 (31.4)	1,548 (29.4)	-
3	1,151 (18.4)	256 (25.4)	895 (17.0)	-
Left main disease	349 (5.6)	92 (9.1)	257 (4.9)	-
LVEF (%), mean, Mean (SD)*	56.73 (12.94)	46.18 (15.00)	58.80 (11.41)	< 0.001
≤40%	241 (12.7)	119 (38.0)	122 (7.7)	< 0.001
Procedural data				
Puncture site				
Radial access only	4,374 (69.8)	570 (56.7)	3,804 (72.3)	< 0.001
Femoral access	1,631 (26.0)	365 (36.3)	1,266 (24.1)	-
Brachial access	269 (4.3)	74 (7.4)	195 (3.7)	-
Radial access	4,517 (72.1)	594 (59.0)	3,923 (74.6)	-
Imaging guided	5,918 (94.4)	946 (94.0)	4,972 (94.5)	0.548
PCI for chronic total occlusion	428 (6.8)	78 (7.8)	350 (6.7)	0.219
Medical status at discharge				
Aspirin	6,148 (98.1)	968 (96.2)	5,180 (98.5)	< 0.001
P2Y12 inhibitor	6,209 (99.1)	986 (98.0)	5,223 (99.3)	< 0.001
Prasugrel	3,924 (62.6)	538 (53.5)	3,386 (64.4)	< 0.001
Clopidogrel	2,223 (35.5)	435 (43.2)	1,788 (34.0)	< 0.001
OAC	621 (9.9)	223 (22.2)	398 (7.6)	< 0.001
Proton pump inhibitor	5,302 (84.6)	858 (85.3)	4,444 (84.5)	0.536
NSAIDs	334 (5.3)	55 (5.5)	279 (5.3)	0.818
Steroids	249 (4.0)	47 (4.7)	202 (3.8)	0.218
Antihyperlipidemic agent	5,406 (86.3)	812 (80.7)	4,594 (87.3)	< 0.001
Modified ARC-HBR				
HBR patients	3,192 (50.9)	755 (75.0)	2,437 (46.3)	< 0.001
Complex PCI	1,279 (20.4)	259 (25.7)	1,020 (19.4)	< 0.001
High platelet reactivity (PRU > 208)	2,229 (35.6)	448 (44.5)	1,781 (33.9)	< 0.001

Data are presented as mean (standard deviation) or n (%) values

*ACS* Acute coronary syndrome, *AF* Atrial fibrillation, *ARC-HBR* Academic Research Consortium for High Bleeding Risk, *CABG* Coronary artery bypass, *eGFR* estimated glomerular filtration rate, *Hb* Haemoglobin, *HF*Heart failure, *LVEF* Left ventricular ejection fraction, *MI* Myocardial infarction, *NSAIDs* Non-steroidal anti-inflammatory drugs, *OAC* Oral anticoagulant, *PCI* Percutaneous coronary intervention, *PRU* P2Y12 Reaction Units, *STEMI* ST-elevation myocardial infarction

*LVEF assessed at baseline is restricted to 1902 participants

### Association of HF with outcomes

**Fig. 1:**
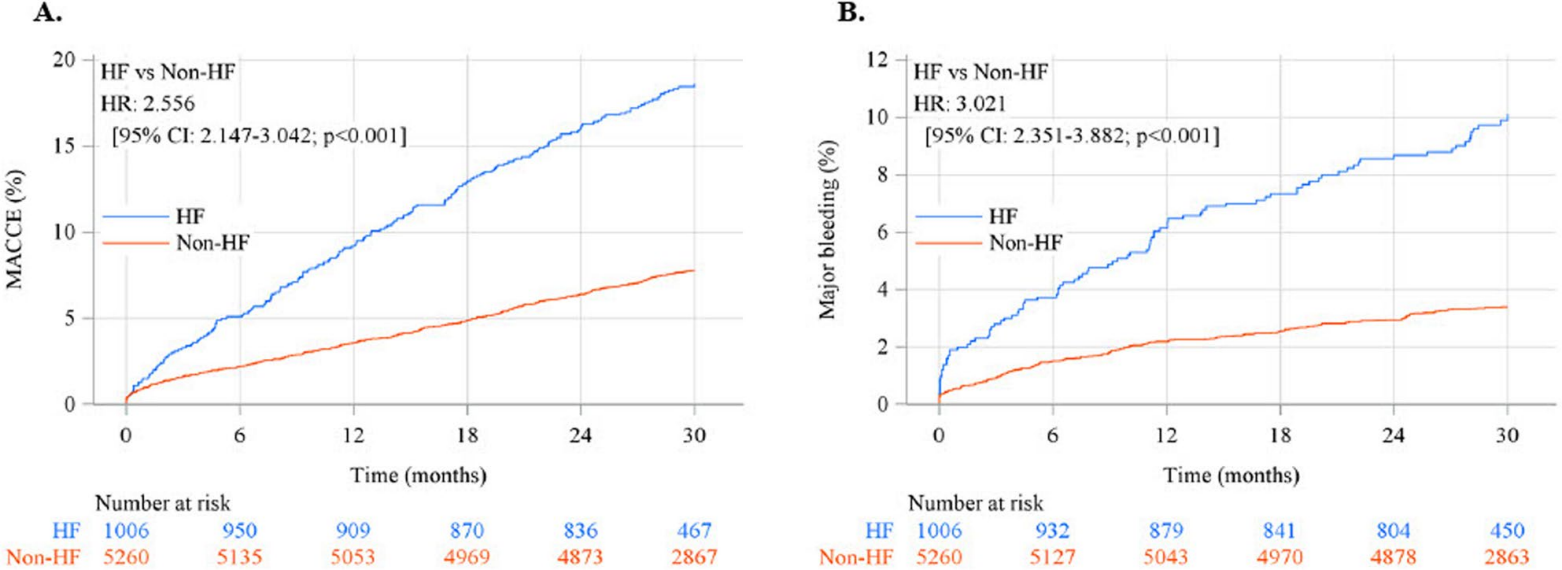
Cumulative incidence of (**A**) major adverse cardiac and cerebrovascular events (MACCE; the standard clinical definition comprising all-cause death, non-fatal MI, non-fatal stroke, and stent thrombosis) and (**B**) major bleeding after percutaneous coronary intervention by with or without heart failure. CI, confidence interval; HF, heart failure; HR, hazard ratio

The risk of MACCE was 2.56 times higher in patients with than in those without HF (Fig. 1A). Cardiac death, noncardiac death, and nonfatal stroke contributed significantly to the higher MACCE rate in HF patients than in non-HF patients. However, there were no significant differences in stent thrombosis and nonfatal MI (Table 2.). After adjustment, HF was still an independent risk factor for MACCE, with an adjusted HR of 1.59. Other identified risk factors for MACCE were male, elderly, low body weight, diabetes mellitus, acute coronary syndrome, PAD, history of stroke, low estimated glomerular filtration rate, and low haemoglobin level (Table 3).

**Table 2:** Cumulative incidence of MACCE, major bleeding, and their components

	Event rate, n (%)			
	HF(n=1006)	Non-HF(n=5260)	HR (95% CI)(Ref: Non-HF)	P value
MACCE [a]	185 (18.4)	403 (7.7)	2.556 (2.147 - 3.042)	<.001
All-cause death	154 (15.3)	249 (4.7)	3.450 (2.822 - 4.217)	<.001
Cardiac death	43 (4.3)	21 (0.4)	11.313 (6.714 - 19.064)	<.001
Non-cardiac death	111 (11.0)	228 (4.3)	2.720 (2.168 - 3.413)	<.001
Non-fatal myocardial infarction	20 (2.0)	81 (1.5)	1.352 (0.829 - 2.206)	0.227
Non-fatal stroke	31 (3.1)	93 (1.8)	1.846 (1.230 - 2.772)	0.003
Stent thrombosis	3 (0.3)	22 (0.4)	0.740 (0.222 - 2.472)	0.625
Major bleeding [b]	94 (9.3)	175 (3.3)	3.021 (2.351 - 3.882)	<.001

*BARC* Bleeding Academic Research Consortium, *CI* Confidence interval, *HF* Heart failure, *HR* Hazard ratio, *MACCE* Major adverse cardiac and cerebrovascular events, *MI* Myocardial infarction

[a] Includes all-cause death, non-fatal myocardial infarction, non-fatal stroke, and stent thrombosis

[b] BARC 3 and 5

**Table 3:** Multivariate adjusted hazard ratios of MACCE and Major bleeding

	n	Events, n (%)	Unadjusted HR (95% CI)	P value	Adjusted HR (95% CI)	P value
MACCE						
HF: yes vs. no	1,006 vs. 5,260	185 (18.4%) vs. 403 (7.7%)	2.513 (2.085 - 3.028)	<.001	1.586 (1.301 - 1.933)	<.001
Sex: female vs. male	1,357 vs. 4,909	127 (9.4%) vs. 461 (9.4%)	0.978 (0.793 - 1.205)	0.831	0.660 (0.522 - 0.835)	<.001
Age: ≥75 vs. <75 y	2,325 vs. 3,941	294 (12.6%) vs. 294 (7.5%)	1.688 (1.423 - 2.004)	<.001	1.222 (1.014 - 1.474)	0.035
Weight: ≤50 vs. >50 kg	794 vs. 5,325	130 (16.4%) vs. 443 (8.3%)	2.061 (1.680 - 2.528)	<.001	1.766 (1.393 - 2.239)	<.001
Diabetes: yes vs. no	2,771 vs. 3,495	324 (11.7%) vs. 264 (7.6%)	1.540 (1.297 - 1.829)	<.001	1.285 (1.075 - 1.537)	0.006
ACS: yes vs. no	2,015 vs. 4,251	203 (10.1%) vs. 385 (9.1%)	1.170 (0.978 - 1.399)	0.085	1.355 (1.131 - 1.624)	<.001
Peripheral arterial disease: yes vs. no	438 vs. 5,828	94 (21.5%) vs. 494 (8.5%)	2.545 (2.003 - 3.233)	<.001	1.435 (1.112 - 1.853)	0.006
History of cerebral infarction or haemorrhage: yes vs. no	619 vs. 5,433	93 (15.0%) vs. 465 (8.6%)	1.821 (1.442 - 2.300)	<.001	1.400 (1.104 - 1.775)	0.006
CKD: eGFR≥30-<60 vs. ≥60 mL/min/1.73 m2	2,093 vs. 3,431	198 (9.5%) vs. 217 (6.3%)	1.546 (1.263 - 1.892)	<.001	1.233 (0.998 - 1.524)	<.001
CKD: eGFR <30 vs. ≥60 mL/min/1.73 m2	597 vs. 3,431	161 (27.0%) vs. 217 (6.3%)	4.904 (3.957 - 6.076)		2.470 (1.905 - 3.204)	
Anaemia: Hb≥11-<13 (M)≥11-<12 (F) vs. ≥13 (M)/≥12 g/dL (F)	1,412 vs. 3,947	169 (12.0%) vs. 237 (6.0%)	2.123 (1.730 - 2.604)	<.001	1.521 (1.223 - 1.891)	<.001
Anaemia: Hb <11 (both) vs. ≥13 (M)/≥12 g/dL (F)	727 vs. 3,947	154 (21.2%) vs. 237 (6.0%)	3.973 (3.223 - 4.898)		1.916 (1.484 - 2.473)	
Major Bleeding						
HF: yes vs. no	1,006 vs. 5,260	94 (9.3%) vs. 175 (3.3%)	2.938 (2.242 - 3.849)	<.001	1.739 (1.300 - 2.325)	<.001
Sex: female vs. male	1,357 vs. 4,909	61 (4.5%) vs. 208 (4.2%)	1.111 (0.822 - 1.502)	0.494	0.723 (0.514 - 1.016)	0.062
Weight: ≤50 vs. >50 kg	794 vs. 5,325	62 (7.8%) vs. 200 (3.8%)	2.395 (1.787 - 3.210)	<.001	2.090 (1.494 - 2.922)	<.001
Anaemia: Hb ≥11-<13 (M)/≥11-<12 (F) vs. ≥13 (M)/≥12 g/dL (F)	1,412 vs. 3,947	68 (4.8%) vs. 119 (3.0%)	1.610 (1.176 - 2.206)	<.001	1.170 (0.845 - 1.619)	0.002
Anaemia: Hb <11 (both) vs. ≥13 (M)/≥12 g/dL (F)	727 vs. 3,947	71 (9.8%) vs. 119 (3.0%)	3.650 (2.694 - 4.946)		1.903 (1.332 - 2.719)	
ACS: yes vs. no	2,015 vs. 4,251	88 (4.4%) vs. 181 (4.3%)	1.153 (0.884 - 1.505)	0.294	1.391 (1.061 - 1.824)	0.017
Peripheral arterial disease: yes vs. no	438 vs. 5,828	39 (8.9%) vs. 230 (3.9%)	2.483 (1.734 - 3.555)	<.001	1.597 (1.093 - 2.333)	0.016
Malignant tumour: yes vs. no	389 vs. 5,877	27 (6.9%) vs. 242 (4.1%)	2.074 (1.380 - 3.116)	<.001	1.699 (1.124 - 2.568)	0.012
Anticoagulation at discharge: yes vs. no	621 vs. 5,645	65 (10.5%) vs. 204 (3.6%)	3.179 (2.372 - 4.260)	<.001	2.628 (1.939 - 3.563)	<.001
CKD: eGFR ≥30-<60 vs. ≥60 mL/min/1.73 m2	2,093 vs. 3,431	105 (5.0%) vs. 98 (2.9%)	1.811 (1.355 - 2.420)	<.001	1.413 (1.045 - 1.910)	<.001
CKD: eGFR <30 vs. ≥60 mL/min/1.73 m2	597 vs. 3,431	62 (10.4%) vs. 98 (2.9%)	4.037 (2.877 - 5.667)		2.119 (1.422 - 3.158)	

*ACS *Acute coronary syndrome*, CI *Confidence interval*, CKD *Chronic kidney disease*, eGFR *estimated glomerular filtration rate*, F *Female*, HR *Hazard ratio*, M *Male*, MACCE *Major adverse cardiovascular and cerebrovascular events*, PCI *Percutaneous coronary intervention

## Effect on outcomes with HBR status by with or without HF

For this analysis, HF and non-HF patients were further stratified into HBR and non-HBR groups (Supplementary Table 1). In common with both HF and non-HF groups, HBR patients were older, underweight, and had more comorbidities such as hypertension, PAD, malignancy, haemodialysis, history of stroke, atrial fibrillation, and more multi-vessel disease than non-HBR patients (Supplementary Table 1). In addition, smoking was rather infrequent, and the rate of ACS was low in HBR patients (Supplementary Table 1).

**Fig. 2:**
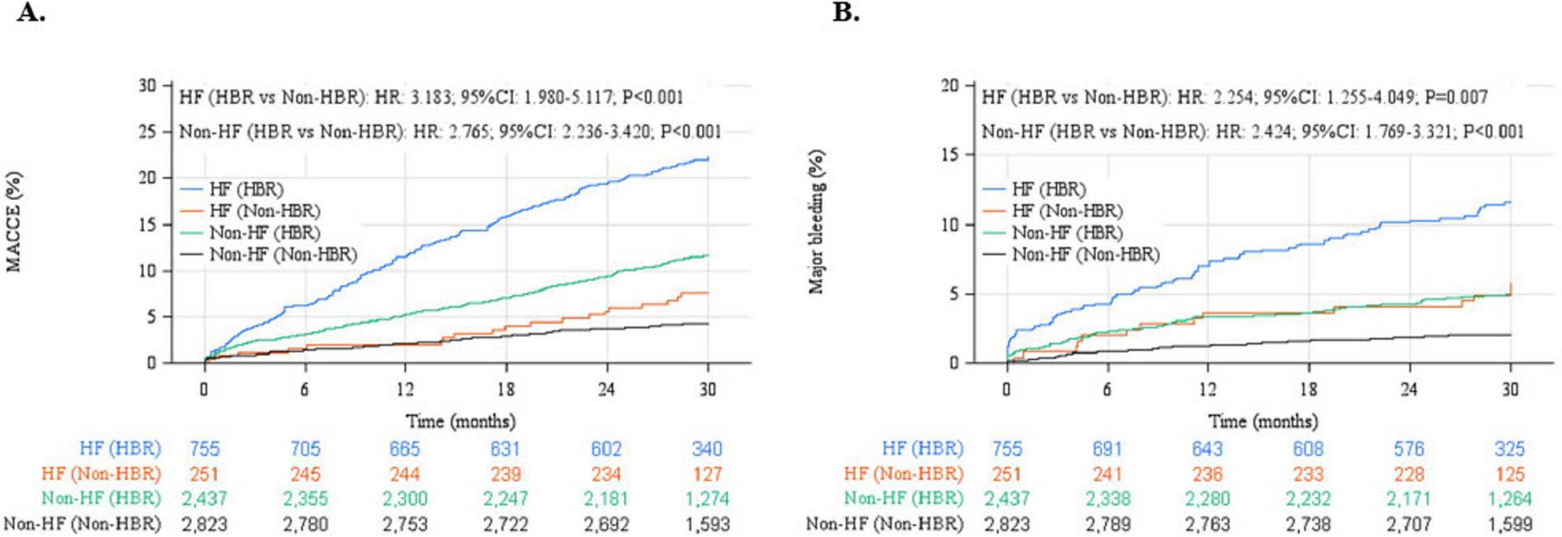
Cumulative incidence of (**A**) major adverse cardiac and cerebrovascular events (MACCE; the standard clinical definition comprising all-cause death, non-fatal MI, non-fatal stroke, and stent thrombosis) and (**B**) major bleeding after percutaneous coronary intervention by with or without heart failure and HBR. CI, confidence interval; HBR, high bleeding risk; HF, heart failure; HR, hazard ratio

The results showed that the incidence of MACCE was about 3.2-fold higher in HBR patients with HF and about 2.8-fold higher in those without HF than in non-HBR patients (Fig. 2A). The rate of MACCE in HF patients without HBR was lower than that of non-HF patients with HBR. The components of MACCE with and without HBR are shown in the supplementary tables (Supplementary Tables 2, 3).

Major bleeding was also about 2.3 to 2.4 times more common in HBR patients compared with non-HBR patients, irrespective of HF (Fig. 2B).

Table 4 shows the outcomes of the Cox proportional hazards models analyses. There was no evidence of interactions with HBR status by the prevalence of HF for MACCE and major bleeding (HR 0.97. 95%CI 0.55–1.70, p for interaction = 0.913, HR 1.31, 95% CI 0.64–2.70, p for interaction 0.456 respectively).

**Table 4:** Multivariate adjusted hazard ratios of MACCE and major bleeding according to HF and HBR

	*n*	Events, *n* (%)	HR (95% CI)	*P* value
MACCE [1]				
HF vs. Non-HF	1,006 vs. 5,260	185 (18.4%) vs. 403 (7.7%)	1.872 (1.524–2.300)	< 0.001
HBR vs. Non-HBR	3,192 vs. 3,074	447 (14.0%) vs. 141 (4.6%)	2.933 (2.330–3.692)	< 0.001
Interaction HF * HBR	-	-	0.969 (0.551–1.705)	0.913
Major bleeding [2]				
HF vs. Non-HF	1,006 vs. 5,260	94 (9.3%) vs. 175 (3.3%)	2.262 (1.236–4.141)	0.008
HBR vs. Non-HBR	3,192 vs. 3,074	198 (6.2%) vs. 71 (2.3%)	2.666 (1.891–3.758)	< 0.001
Interaction HF * HBR	-	-	1.309 (0.636–2.694)	0.465

*ACS* Acute coronary syndrome, *CI* Confidence interval, *HF* Heart failure, *HR* Hazard ratio, *MACCE* Major adverse cardiac and cerebrovascular events, *PAD* Peripheral arterial disease

[1] multivariable Cox proportional hazards model: adjusted with sex, DM, ACS, PAD

[2] multivariable Cox proportional hazards model: adjusted with sex, ACS, PAD

## Discussion

The present study showed that (1) after PCI, HF patients are at significantly higher risk of MACCE and major bleeding events, (2) HBR is associated with higher MACCE and major bleeding irrespective of HF and non-HF patients, and (3) both HF and HBR are independent determinants of MACCE and major bleeding. Therefore, this study suggests that each was independently associated with prognosis and should be considered in management.

There were significantly more MACCE (2.56 folds) and major bleeding events (3.02 folds) in HF patients after PCI. Furthermore, even after adjustment, HF was a risk factor for both MACCE and major bleeding. The American College of Cardiology/American Heart Association guidelines also reported that a history of congestive HF is a high-risk condition for cardiovascular events [16]. HF creates a hypercoagulable state that is associated with adverse events, including stroke, systemic embolism, and mortality [17–20]. Interestingly, in the present study, HPR, suggesting ineffectiveness of antiplatelet agents, was observed more frequently in HF patients. This may be due to the elevated levels of pro-thrombotic and pro-inflammatory cytokines observed in patients with HF. Thus, inadequate antiplatelet action may be the cause of the higher MACCE rate in HF patients. All other identified risk factors for MACCE in this study were consistent with previous reports and well-known factors [8, 21, 22]. The majority of the identified factors were components of the ARC-HBR in which consensus agreement was achieved. Further, HF was suggested to be an independent factor for major bleeding [22]. Previous studies reported incidence rates of bleeding events ranging from 8.0% to 12.9% in patients undergoing PCI with HF. [23, 24] The incidence rate of major bleeding events in HF patients after PCI was 9.3% in the present study, which was consistent with the results of previous studies. Thus, the risk of major bleeding after PCI for patients with HF is likely to be extremely high, because ARC defined HBR as an annual major bleeding event rate > 4%. The association between the pathophysiology of HF and bleeding has been mentioned to involve low cardiac output and increased central venous pressure due to congestive heart failure causing abnormal intestinal function, which induces bleeding from the mucosa of the gastrointestinal tract [25]. In addition, heart failure patients after PCI needed to receive antiplatelet therapy, further increasing the risk of bleeding, including gastrointestinal bleeding [24]. Another explanation is that HF frequently coexists with many features included in the HBR criteria and is similarly associated with multiple comorbidities, concomitant medications, and a higher rate of hospital readmissions, etc. In Japan, the impact of HF may be more pronounced than in other countries. It has been reported that many HF patients in Japan are underweight and frail, whereas many HF patients overseas are obese. Indeed, in the present study, the HF group had significantly lower weight than the non-HF group. In addition, the HF group had significantly lower Hb and more frequent anaemia than the non-HF group.

HF increased the risk of MACCE and major bleeding, and HBR increased the risk of MACCE and major bleeding regardless of HF. Since the present study found no interaction in the relationship between HF and HBR for MACCE and major bleeding, HF and HBR are likely to be independently related to prognosis. The wide confidence intervals indicate limited power for the interaction analyses; nonsignificant results should not be taken as evidence of equivalence. Conclusions on the independence of HF and HBR effects remain tentative, and larger studies are needed to confirm potential effect modification. A previous study reported that patients with bleeding events after PCI had significantly higher rates of cardiovascular events [26, 27]. In addition, there is a report that bleeding events in acute decompensated HF were associated with HF re-hospitalization [28]. In this study, many patient characteristics overlapped between HBR and HF, suggesting that the observed association between bleeding and cardiovascular events may reflect shared underlying mechanisms. However, this interpretation remains speculative, as no temporal analyses were conducted to formally support a causal or sequential relationship. The plausible mechanisms of bleeding events with subsequent mortality include activation of the coagulation cascade, increased prothrombotic cytokine levels, hypovolemia, anaemia, reflex tachycardia, transfusion of blood products, and cessation of antiplatelet and anticoagulant therapies [26]. In particular, as a potential mechanism underlying bleeding-induced inflammation and prothrombotic states, it has been demonstrated that the hemostatic process following bleeding can trigger inflammation, which in turn serves as a potent prothrombotic stimulus by activating platelets and the coagulation cascade [29]. 

### Clinical implications

In the present study, HF patients had more atrial fibrillation and anticoagulants than non-HF patients, resulting in high rates of bleeding, as well as a high percentage of patients with low EF and undergoing complex PCI and a high risk of cardiovascular thrombotic events.

That the P2Y12-infibitor monotherapy reduced bleeding events without increasing ischemic events in patients undergoing complex PCI was reported [30]. In this study, nearly all patients were discharged on dual antiplatelet therapy, suggesting that subsequent transition to P2Y12 inhibitor monotherapy may contribute to improved clinical outcomes.

Therefore, optimizing the balance between ischemic and bleeding events is important for HF patients undergoing PCI, such as those included in this study, especially those with AF. Careful risk management during both the peri-PCI and post-PCI phases is essential to improve outcomes in AF patients undergoing PCI [31]. For example, procedural strategies using intracoronary imaging, the appropriate selection of antithrombotic agents, and the tailoring of treatment duration are critical considerations. Moreover, the safety and efficacy of direct oral anticoagulant monotherapy as an antithrombotic strategy for patients with HF, coronary artery disease, and AF have been reported, suggesting that such an approach may help reduce both bleeding and cardiovascular events in the high-risk population represented in the present study [32]. 

Furthermore, HF patients underwent complex PCI more frequently than non-HF patients, and consequently the use of femoral artery access was also higher in this study. These findings suggest that access site selection may have contributed to the increased incidence of bleeding events following PCI. The importance of selecting an access site that minimizes periprocedural bleeding complications during PCI has been well recognized. [33] Procedural characteristics differed substantially between HF and non-HF groups, and only limited adjustment was feasible, which may have influenced bleeding comparisons. Unfortunately, further stratified analyses were not possible with the available data. Future studies are warranted to clarify the impact of procedural strategies in this high-risk population.

In summary, patients with factors of HF and HBR after PCI should be considered more important in daily clinical practice to improve their prognosis. In addition, we should pay attention to the occurrence of major bleeding, as well as MACCE, after PCI for patients with HF. Although HF is not widely recognized as a risk factor for major bleeding, major bleeding may be a cause of subsequent MACCE. Therefore, such patients should be recognized as a high-risk population, warranting long-term risk monitoring and frequent follow-up. In addition, careful consideration of the optimal antithrombotic therapy for each individual patient is essential. In the future, larger studies aimed at reducing the risk of MACCE and bleeding events in a general population of HF patients after PCI seem warranted.

### Study limitations

There are several limitations to the present study. This analysis used data from the PENDULUM registry and was conducted in a post-hoc manner, meaning that the absence of prespecified hypotheses and statistical models entails inherent risks of multiplicity and analytical bias. As such, causal inferences should be made with caution, and the results may be subject to residual or unmeasured confounding. This is particularly relevant because both HF and bleeding outcomes are influenced by various clinical, procedural, and therapeutic factors not fully captured in our data. In addition, although residual and unmeasured confounding factors could not be included in the Cox model, limiting the interpretation of our findings, the present study provides valuable insights into the outcomes of HF patients undergoing PCI. Future hypothesis-driven investigations in well-defined HF cohorts are needed to confirm these findings and strengthen the evidence for clinical practice. Further, information on the New York Heart Association functional class, Brain natriuretic peptide, a presence of prior hospitalization, and adverse events related with HF was lacking. As NT-proBNP measurements and echocardiographic data were unavailable, misclassification between HF with reduced and preserved ejection fraction cannot be excluded. Such nondifferential misclassification would likely bias associations toward the null, while the under-recognition of milder HF may overestimate risk among identified HF patients. Although history-based identification is clinically straightforward, the absence of standardized diagnostic criteria limits the interpretation of our findings. Additionally, a substantial proportion of important variables, including left ventricular EF, were missing in this registry. Imputation was not performed due to the high rate of missingness. This limitation may introduce bias and reduce the precision of the estimates, and therefore the results should be interpreted with caution. The causal relationship between HF and bleeding events was unclear. Because the PENDULUM study was not designed as a dedicated heart failure registry, detailed information regarding HF medications and treatment trajectories was not systematically collected. As a result, our ability to assess treatment effects or derive specific management implications is limited. The study population consisted entirely of Japanese patients, who exhibit the “East Asian paradox” of lower thrombotic and higher bleeding risk. [34] Consequently, the thrombotic and bleeding profiles may differ from Western populations, limiting the generalizability of our findings. Nonetheless, as previously reported, the relationship between platelet function and thrombotic events observed in ADAPT-DES [14] appears to be consistent with that in the PENDULUM study [7, 8]. 

## Conclusions

In patients after contemporary PCI, HF and HBR were independently associated with a higher risk of MACCE and bleeding. De-escalation management of antithrombotic drugs that prevent major bleeding and reduce subsequent cardiovascular events is justified in HF and HBR cases. Future studies should focus on validating these results in a more generalized population of HF patients after PCI. There is also a need for prospective studies on this topic.

### Data Availability

The datasets are available from the corresponding author on reasonable request.

## Supplementary Information

Supplementary Material 1.

## Data Availability

The datasets are available from the corresponding author on reasonable request.

## Declarations

### Clinical trial number

UMIN000020332　(URL: https://www.umin.ac.jp/ctr/).
