# Supplementary material for: The impact of heart failure and bleeding risk on clinical outcomes in patients after percutaneous coronary intervention
Source: BMC Cardiovasc Disord. 2025 Dec 19;26:70. doi: 10.1186/s12872-025-05429-6 (PMC12831353; doi:10.1186/s12872-025-05429-6)
Supplement: Supplementary file 1 — Supplementary Material 1. [file 12872_2025_5429_MOESM1_ESM.docx]

**Supplementary materials**

**Supplementary Table 1**. Baseline characteristics by with or without HF and HBR

|  | Total | HF | | | Non-HF | | |
| --- | --- | --- | --- | --- | --- | --- | --- |
|  | (n=6,266) | HBR (n=755) | Non-HBR (n=251) | P value | HBR (n=2,437) | Non-HBR (n=2,823) | P value |
| Age (y), mean (SD) | 70.0 (10.7) | 75.3 (10.1) | 65.1 (10.5) | <.001 | 74.7 (8.9) | 65.0 (9.8) | <.001 |
| ≥75 years | 2,325 (37.1) | 458 (60.7) | 39 (15.5) | <.001 | 1,469 (60.3) | 359 (12.7) | <.001 |
| Body weight (kg), mean (SD) | 63.9 (12.6) | 59.3 (12.4) | 67.5 (13.7) | <.001 | 61.3 (11.9) | 67.1 (12.3) | <.001 |
| ≤50 kg | 794 (12.7) | 169 (22.4) | 20 (8.0) | <.001 | 414 (17.0) | 191 (6.8) | <.001 |
| Body mass index (kg/m^2^), mean (SD) | 24.21 (3.62) | 23.29 (3.77) | 24.98 (4.24) | <.001 | 23.77 (3.50) | 24.77 (3.51) | <.001 |
| Hypertension | 5,188 (82.8) | 685 (90.7) | 210 (83.7) | 0.003 | 2,095 (86.0) | 2,198 (77.9) | <.001 |
| Hyperlipidaemia | 4,926 (78.6) | 558 (73.9) | 204 (81.3) | 0.022 | 1,851 (76.0) | 2,313 (81.9) | <.001 |
| Diabetes mellitus | 2,771 (44.2) | 420 (55.6) | 147 (58.6) | 0.420 | 1,102 (45.2) | 1,102 (39.0) | <.001 |
| Current cigarette smoking | 1,327 (21.2) | 106 (14.0) | 75 (29.9) | <.001 | 347 (14.2) | 799 (28.3) | <.001 |
| Peripheral arterial disease | 438 (7.0) | 100 (13.2) | 6 (2.4) | <.001 | 233 (9.6) | 99 (3.5) | <.001 |
| AF | 539 (8.6) | 183 (24.2) | 9 (3.6) | <.001 | 297 (12.2) | 50 (1.8) | <.001 |
| Malignancy | 389 (6.2) | 78 (10.3) | 0 | <.001 | 311 (12.8) | 0 | <.001 |
| Previous MI | 1,575 (25.1) | 288 (38.1) | 106 (42.2) | 0.263 | 538 (22.1) | 643 (22.8) | 0.551 |
| Previous PCI | 2,566 (41.0) | 347 (46.0) | 123 (49.0) | 0.422 | 1,014 (41.6) | 1,082 (38.3) | 0.016 |
| Previous CABG | 265 (4.2) | 70 (9.3) | 15 (6.0) | 0.116 | 109 (4.5) | 71 (2.5) | <.001 |
| History of ischaemic stroke | 657 (10.5) | 139 (18.4) | 14 (5.6) | <.001 | 420 (17.2) | 84 (3.0) | <.001 |
| History of cerebral haemorrhage | 124 (2.0) | 25 (3.3) | 0 | 0.001 | 99 (4.1) | 0 | <.001 |
| History of renal insufficiency | 1,114 (17.8) | 329 (43.6) | 17 (6.8) | <.001 | 612 (25.1) | 156 (5.5) | <.001 |
| Clinical presentation |  |  |  |  |  |  |  |
| ACS | 2,015 (32.2) | 189 (25.0) | 72 (28.7) | 0.280 | 735 (30.2) | 1,019 (36.1) | <.001 |
| Unstable angina | 790 (12.6) | 63 (8.3) | 20 (8.0) | 0.003 | 324 (13.3) | 383 (13.6) | 0.007 |
| Non-STEMI | 323 (5.2) | 51 (6.8) | 8 (3.2) | - | 114 (4.7) | 150 (5.3) | - |
| STEMI | 908 (14.5) | 75 (9.9) | 44 (17.5) | - | 299 (12.3) | 490 (17.4) | - |
| Hb (g/dL), mean (SD) | 13.30 (2.04) | 11.83 (2.04) | 14.27 (2.75) | <.001 | 12.42 (1.78) | 14.36 (1.50) | <.001 |
| Hb: <11 g/dL (both) | 727 (11.6) | 250 (33.1) | 0 | <.001 | 477 (19.6) | 0 | <.001 |
| eGFR (mL/min/1.73 m^2^), mean (SD) | 61.25 (27.61) | 42.88 (23.47) | 73.50 (46.39) | <.001 | 51.81 (23.15) | 73.41 (23.78) | <.001 |
| eGFR: <60 mL/min/1.73 m^2^ | 2,690 (42.9) | 577 (76.4) | 54 (21.5) | <.001 | 1,597 (65.5) | 462 (16.4) | <.001 |
| Angiographic features |  |  |  | - |  |  | - |
| No. of diseased vessels |  |  |  | - |  |  | - |
| 1 | 3,165 (50.5) | 300 (39.7) | 119 (47.4) | 0.034 | 1,178 (48.3) | 1,568 (55.5) | <.001 |
| 2 | 1,864 (29.7) | 237 (31.4) | 79 (31.5) | - | 754 (30.9) | 794 (28.1) | - |
| 3 | 1,151 (18.4) | 206 (27.3) | 50 (19.9) | - | 475 (19.5) | 420 (14.9) | - |
| Left main disease | 349 (5.6) | 74 (9.8) | 18 (7.2) | - | 129 (5.3) | 128 (4.5) | - |
| LVEF (%), mean, Mean (SD) | 56.73 (12.94) | 45.20 (14.98) | 48.77 (14.82) | 0.060 | 58.49 (11.97) | 59.06 (10.91) | 0.321 |
| ≤40% | 241 (12.7) | 90 (39.6) | 29 (33.7) | 0.363 | 66 (9.1) | 56 (6.5) | 0.058 |
| Procedural data |  |  |  |  |  |  |  |
| Puncture site |  |  |  |  |  |  |  |
| Radial access only | 4,374 (69.8) | 408 (54.0) | 162 (64.5) | 0.004 | 1,627 (66.8) | 2,177 (77.1) | <.001 |
| Femoral access | 1,631 (26.0) | 287 (38.0) | 78 (31.1) | - | 699 (28.7) | 567 (20.1) | - |
| Brachial access | 269 (4.3) | 62 (8.2) | 12 (4.8) | - | 114 (4.7) | 81 (2.9) | - |
| Radial access | 4,517 (72.1) | 421 (55.8) | 173 (68.9) | - | 1,670 (68.5) | 2,253 (79.8) | - |
| Imaging guided | 5,918 (94.4) | 709 (93.9) | 237 (94.4) | 0.878 | 2,298 (94.3) | 2,674 (94.7) | 0.504 |
| PCI for chronic total occlusion | 428 (6.8) | 49 (6.5) | 29 (11.6) | 0.014 | 155 (6.4) | 195 (6.9) | 0.438 |
| Medical status at discharge |  |  |  |  |  |  |  |
| Aspirin | 6,148 (98.1) | 723 (95.8) | 245 (97.6) | 0.251 | 2,379 (97.6) | 2,801 (99.2) | <.001 |
| P2Y12 inhibitor | 6,209 (99.1) | 736 (97.5) | 250 (99.6) | 0.036 | 2,407 (98.8) | 2,816 (99.8) | <.001 |
| Prasugrel | 3,924 (62.6) | 366 (48.5) | 172 (68.5) | <.001 | 1,396 (57.3) | 1,990 (70.5) | <.001 |
| Clopidogrel | 2,223 (35.5) | 358 (47.4) | 77 (30.7) | <.001 | 984 (40.4) | 804 (28.5) | <.001 |
| OAC | 621 (9.9) | 223 (29.5) | 0 | <.001 | 398 (16.3) | 0 | <.001 |
| Proton pump inhibitor | 5,302 (84.6) | 641 (84.9) | 217 (86.5) | 0.607 | 2,048 (84.0) | 2,396 (84.9) | 0.423 |
| NSAIDs | 334 (5.3) | 52 (6.9) | 3 (1.2) | <.001 | 207 (8.5) | 72 (2.6) | <.001 |
| Steroids | 249 (4.0) | 44 (5.8) | 3 (1.2) | 0.002 | 153 (6.3) | 49 (1.7) | <.001 |
| Antihyperlipidaemic agent | 5,406 (86.3) | 593 (78.5) | 219 (87.3) | 0.002 | 2,012 (82.6) | 2,582 (91.5) | <.001 |
| Modified ARC-HBR |  |  |  |  |  |  |  |
| HBR patients | 0 | 755 (100.0) | 0 | <.001 | 2,437 (100.0) | 0 | - |
| Complex PCI | 1,279 (20.4) | 190 (25.2) | 69 (27.5) | 0.505 | 488 (20.0) | 532 (18.8) | 0.294 |
| High platelet reactivity (PRU>208) | 2,229 (35.6) | 373 (49.4) | 75 (29.9) | <.001 | 1,041 (42.7) | 740 (26.2) | <.001 |

Data are presented as mean (standard deviation) or n (%) values.

P-values were calculated using Student's *t*-test for continuous variables and Fisher's exact test for categorical variables.

ACS, acute coronary syndrome; AF, atrial fibrillation; CABG, coronary artery bypass; eGFR, estimated glomerular filtration rate; Hb, haemoglobin; HBR, high bleeding risk; HF, heart failure; LVEF, left ventricular ejection fraction; MI, myocardial infarction; NSAIDs, non-steroidal anti-inflammatory drugs; OAC, oral anticoagulant; PCI, percutaneous coronary intervention; PRU, P2Y12 Reaction Units; STEMI, ST-elevation myocardial infarction.

**Supplementary Table 2**. Cumulative incidence of each study endpoint in HF patients

|  | Event rate, n (%) | |  | |
| --- | --- | --- | --- | --- |
|  | HBR (n=755) | Non-HBR (n=251) | HR (95% CI) | P value |
| MACCE [a] | 166 (22.0) | 19 (7.6) | 3.183 (1.980 - 5.117) | <.001 |
| All-cause death | 140 (18.5) | 14 (5.6) | 3.587 (2.071 - 6.213) | <.001 |
| Cardiac death | 37 (4.9) | 6 (2.4) | 2.199 (0.928 - 5.211) | 0.073 |
| Non-cardiac death | 103 (13.6) | 8 (3.2) | 4.628 (2.254 - 9.503) | <.001 |
| Non-fatal myocardial infarction | 18 (2.4) | 2 (0.8) | 3.170 (0.736 - 13.660) | 0.122 |
| Non-fatal stroke | 28 (3.7) | 3 (1.2) | 3.355 (1.020 - 11.034) | 0.046 |
| Stent thrombosis | 3 (0.4) | 0 | - (0.000 - .) | 0.995 |
| Major bleeding [b] | 81 (10.7) | 13 (5.2) | 2.254 (1.255 - 4.049) | 0.007 |

BARC, Bleeding Academic Research Consortium; CI, confidence interval; HR, hazard ratio; MACCE, major adverse cardiac and cerebrovascular events; HBR, high bleeding risk; HF, heart failure.

[a] Includes all-cause death, non-fatal myocardial infarction, non-fatal stroke, and stent thrombosis.

[b] BARC 3 and 5.

**Supplementary Table 3**. Cumulative incidence of each study endpoint in non HF patients

|  | Event rate, n (%) | |  | |
| --- | --- | --- | --- | --- |
|  | HBR (n=2437) | Non-HBR (n=2823) | HR (95% CI) | P value |
| MACCE [a] | 281 (11.5) | 122 (4.3) | 2.765 (2.236 - 3.420) | <.001 |
| All-cause death | 195 (8.0) | 54 (1.9) | 4.327 (3.201 - 5.849) | <.001 |
| Cardiac death | 20 (0.8) | 1 (0.0) | 23.824 (3.197 - 177.519) | 0.002 |
| Non-Cardiac death | 175 (7.2) | 53 (1.9) | 3.959 (2.911 - 5.383) | <.001 |
| Non-fatal myocardial infarction | 48 (2.0) | 33 (1.2) | 1.730 (1.111 - 2.696) | 0.015 |
| Non-fatal stroke | 54 (2.2) | 39 (1.4) | 1.652 (1.094 - 2.494) | 0.017 |
| Stent thrombosis | 13 (0.5) | 9 (0.3) | 1.708 (0.730 - 3.996) | 0.217 |
| Major bleeding [b] | 117 (4.8) | 58 (2.1) | 2.424 (1.769 - 3.321) | <.001 |

BARC, Bleeding Academic Research Consortium; CI, confidence interval; HR, hazard ratio; MACCE, major adverse cardiac and cerebrovascular events; HBR, High Bleeding Risk; HF, heart failure.

[a] Includes all-cause death, non-fatal myocardial infarction, non-fatal stroke, and stent thrombosis.

[b] BARC 3 and 5.
